# Supplementary material for: Life in the fastlane? A comparative analysis of gene expression profiles across annual, semi-annual, and non-annual killifishes (Cyprinodontiformes: Nothobranchiidae)
Source: PLoS One. 2024 Sep 10;19(9):e0308855. doi: 10.1371/journal.pone.0308855 (PMC11386455; doi:10.1371/journal.pone.0308855)
Supplement: S1 Table — (DOCX) [file pone.0308855.s001.docx]

**S1 Table.** Number of DEGs submitted and filtered out in the DAVID webserver.

| **Life History comparisons** | **Official gene symbols** | **Not convertible in DAVID database** | **Entrez IDs submitted to DAVID** |
| --- | --- | --- | --- |
| Up regulated Annuals vs Non-annuals | 3731 | 149 | 3582 |
| Down regulated Annuals vs Non-annuals | 860 | 352 | 508 |
| Up regulated Annuals vs Semi-annuals | 2002 | 568 | 1434 |
| Down regulated Annuals vs Semi-annuals | 343 | 123 | 220 |
| Up regulated Semi-annuals vs Non-annuals | 651 | 172 | 479 |
| Down regulated Semi-annuals vs Non-annuals | 536 | 192 | 344 |
