# Supplementary material for: Life in the fastlane? A comparative analysis of gene expression profiles across annual, semi-annual, and non-annual killifishes (Cyprinodontiformes: Nothobranchiidae)
Source: PLoS One. 2024 Sep 10;19(9):e0308855. doi: 10.1371/journal.pone.0308855 (PMC11386455; doi:10.1371/journal.pone.0308855)
Supplement: S2 Table — Enriched pathways obtained from submitting the DEGs to DAVID webserver. Threshold of minimum gene counts 2 (belonging to an annotation term) and EASE score threshold 0.05 were used to determine significant KEGG pathways. (DOCX) [file pone.0308855.s002.docx]

**S2 Table.** KEGG: annuals vs. non-annuals (liver). Enriched pathways obtained from submitting the DEGs to DAVID webserver. Threshold of minimum gene counts 2 (belonging to an annotation term) and EASE score threshold 0.05 were used to determine significant KEGG pathways.

| **Term** | **Gene count** | **% from DEGs** | **PValue** |
| --- | --- | --- | --- |
| nfu01100:Metabolic pathways | 395 | 11.027 | 3.11E-19 |
| nfu00900:Terpenoid backbone biosynthesis | 14 | 0.391 | 4.21E-06 |
| nfu00280:Valine, leucine and isoleucine degradation | 22 | 0.614 | 1.61E-05 |
| nfu00983:Drug metabolism - other enzymes | 25 | 0.698 | 1.82E-05 |
| nfu00520:Amino sugar and nucleotide sugar metabolism | 22 | 0.614 | 1.44E-04 |
| nfu00240:Pyrimidine metabolism | 23 | 0.642 | 3.00E-04 |
| nfu00400:Phenylalanine, tyrosine and tryptophan biosynthesis | 7 | 0.195 | 3.16E-04 |
| nfu01240:Biosynthesis of cofactors | 45 | 1.256 | 3.43E-04 |
| nfu00982:Drug metabolism - cytochrome P450 | 16 | 0.447 | 6.73E-04 |
| nfu04146:Peroxisome | 28 | 0.782 | 1.15E-03 |
| nfu01232:Nucleotide metabolism | 30 | 0.838 | 1.27E-03 |
| nfu01200:Carbon metabolism | 35 | 0.977 | 1.90E-03 |
| nfu01250:Biosynthesis of nucleotide sugars | 16 | 0.447 | 2.07E-03 |
| nfu00071:Fatty acid degradation | 16 | 0.447 | 2.67E-03 |
| nfu00380:Tryptophan metabolism | 17 | 0.475 | 3.12E-03 |
| nfu00330:Arginine and proline metabolism | 18 | 0.503 | 3.53E-03 |
| nfu00980:Metabolism of xenobiotics by cytochrome P450 | 14 | 0.391 | 3.91E-03 |
| nfu00790:Folate biosynthesis | 12 | 0.335 | 5.66E-03 |
| nfu04115:p53 signaling pathway | 25 | 0.698 | 6.08E-03 |
| nfu00620:Pyruvate metabolism | 16 | 0.447 | 6.59E-03 |
| nfu00020:Citrate cycle (TCA cycle) | 12 | 0.335 | 1.22E-02 |
| nfu00040:Pentose and glucuronate interconversions | 11 | 0.307 | 1.33E-02 |
| nfu03030:DNA replication | 13 | 0.363 | 1.38E-02 |
| nfu00190:Oxidative phosphorylation | 33 | 0.921 | 1.48E-02 |
| nfu04142:Lysosome | 39 | 1.089 | 1.50E-02 |
| nfu00970:Aminoacyl-tRNA biosynthesis | 14 | 0.391 | 1.51E-02 |
| nfu00360:Phenylalanine metabolism | 8 | 0.223 | 1.52E-02 |
| nfu00860:Porphyrin metabolism | 12 | 0.335 | 1.53E-02 |
| nfu00564:Glycerophospholipid metabolism | 28 | 0.782 | 2.08E-02 |
| nfu00410:beta-Alanine metabolism | 11 | 0.307 | 2.12E-02 |
| nfu00053:Ascorbate and aldarate metabolism | 10 | 0.279 | 2.35E-02 |
| nfu04621:NOD-like receptor signaling pathway | 36 | 1.005 | 2.60E-02 |
| nfu03020:RNA polymerase | 11 | 0.307 | 2.63E-02 |
| nfu04145:Phagosome | 36 | 1.005 | 2.83E-02 |
| nfu04110:Cell cycle | 33 | 0.921 | 3.45E-02 |
| nfu00600:Sphingolipid metabolism | 19 | 0.530 | 4.01E-02 |
| nfu00640:Propanoate metabolism | 10 | 0.279 | 4.44E-02 |
| nfu00100:Steroid biosynthesis | 8 | 0.223 | 4.62E-02 |
| nfu00561:Glycerolipid metabolism | 19 | 0.530 | 5.05E-02 |
| nfu00010:Glycolysis / Gluconeogenesis | 19 | 0.530 | 5.05E-02 |
| nfu04216:Ferroptosis | 15 | 0.419 | 5.50E-02 |
| nfu00480:Glutathione metabolism | 17 | 0.475 | 5.97E-02 |
| nfu00591:Linoleic acid metabolism | 7 | 0.195 | 6.53E-02 |
| nfu00650:Butanoate metabolism | 7 | 0.195 | 6.53E-02 |
| nfu00590:Arachidonic acid metabolism | 14 | 0.391 | 7.22E-02 |
| nfu00770:Pantothenate and CoA biosynthesis | 7 | 0.195 | 9.94E-02 |
| nfu01210:2-Oxocarboxylic acid metabolism | 7 | 0.195 | 9.94E-02 |
