# Supplementary material for: Life in the fastlane? A comparative analysis of gene expression profiles across annual, semi-annual, and non-annual killifishes (Cyprinodontiformes: Nothobranchiidae)
Source: PLoS One. 2024 Sep 10;19(9):e0308855. doi: 10.1371/journal.pone.0308855 (PMC11386455; doi:10.1371/journal.pone.0308855)
Supplement: S3 Table — Enriched pathways obtained from submitting the DEGs to DAVID webserver. Threshold of minimum gene counts 2 (belonging to an annotation term) and EASE score threshold 0.05 were used to determine significant KEGG pathways. (DOCX) [file pone.0308855.s003.docx]

**S3 Table.** KEGG: annuals vs. semi-annuals (liver). Enriched pathways obtained from submitting the DEGs to DAVID webserver. Threshold of minimum gene counts 2 (belonging to an

annotation term) and EASE score threshold 0.05 were used to determine significant KEGG pathways.

| **Term** | **Count** | **% from DEGs** | **PValue** |
| --- | --- | --- | --- |
| nfu01100:Metabolic pathways | 199 | 10.056 | 1.582E-08 |
| nfu00983:Drug metabolism - other enzymes | 18 | 0.910 | 8.361E-06 |
| nfu00860:Porphyrin metabolism | 12 | 0.606 | 6.238E-05 |
| nfu01240:Biosynthesis of cofactors | 30 | 1.516 | 7.912E-05 |
| nfu00040:Pentose and glucuronate interconversions | 10 | 0.505 | 4.324E-04 |
| nfu04145:Phagosome | 27 | 1.364 | 5.439E-04 |
| nfu00982:Drug metabolism - cytochrome P450 | 11 | 0.556 | 1.034E-03 |
| nfu00053:Ascorbate and aldarate metabolism | 9 | 0.455 | 1.288E-03 |
| nfu00120:Primary bile acid biosynthesis | 7 | 0.354 | 6.712E-03 |
| nfu00480:Glutathione metabolism | 13 | 0.657 | 7.823E-03 |
| nfu00980:Metabolism of xenobiotics by cytochrome P450 | 9 | 0.455 | 9.865E-03 |
| nfu03008:Ribosome biogenesis in eukaryotes | 14 | 0.707 | 1.040E-02 |
| nfu04142:Lysosome | 24 | 1.213 | 1.068E-02 |
| nfu00140:Steroid hormone biosynthesis | 10 | 0.505 | 1.999E-02 |
| nfu04623:Cytosolic DNA-sensing pathway | 9 | 0.455 | 2.049E-02 |
| nfu00280:Valine, leucine and isoleucine degradation | 10 | 0.505 | 2.250E-02 |
| nfu00630:Glyoxylate and dicarboxylate metabolism | 8 | 0.404 | 3.085E-02 |
| nfu00240:Pyrimidine metabolism | 11 | 0.556 | 3.866E-02 |
| nfu04146:Peroxisome | 14 | 0.707 | 4.221E-02 |
| nfu01232:Nucleotide metabolism | 15 | 0.758 | 4.341E-02 |
| nfu00380:Tryptophan metabolism | 9 | 0.455 | 4.626E-02 |
| nfu04210:Apoptosis | 22 | 1.112 | 4.891E-02 |
