# Supplementary material for: Life in the fastlane? A comparative analysis of gene expression profiles across annual, semi-annual, and non-annual killifishes (Cyprinodontiformes: Nothobranchiidae)
Source: PLoS One. 2024 Sep 10;19(9):e0308855. doi: 10.1371/journal.pone.0308855 (PMC11386455; doi:10.1371/journal.pone.0308855)
Supplement: S4 Table — Enriched pathways obtained from submitting the DEGs to DAVID webserver. Threshold of minimum gene counts 2 (belonging to an annotation term) and EASE score threshold 0.05 were used to determine significant KEGG pathways. (DOCX) [file pone.0308855.s004.docx]

**S4 Table.** KEGG: semi-annuals vs. non-annuals (liver). Enriched pathways obtained from submitting the DEGs to DAVID webserver. Threshold of minimum gene counts 2 (belonging to an annotation term) and EASE score threshold 0.05 were used to determine significant KEGG pathways.

| **Term** | **Count** | **%** | **PValue** |
| --- | --- | --- | --- |
| nfu01100:Metabolic pathways | 84 | 12.766 | 1.71E-05 |
| nfu00190:Oxidative phosphorylation | 13 | 1.976 | 1.47E-03 |
| nfu01200:Carbon metabolism | 11 | 1.672 | 8.98E-03 |
| nfu00071:Fatty acid degradation | 6 | 0.912 | 1.34E-02 |
| nfu00510:N-Glycan biosynthesis | 7 | 1.064 | 1.66E-02 |
| nfu00360:Phenylalanine metabolism | 4 | 0.608 | 1.81E-02 |
| nfu00513:Various types of N-glycan biosynthesis | 6 | 0.912 | 2.38E-02 |
| nfu00630:Glyoxylate and dicarboxylate metabolism | 5 | 0.760 | 3.13E-02 |
| nfu00640:Propanoate metabolism | 4 | 0.608 | 7.38E-02 |
| nfu01212:Fatty acid metabolism | 6 | 0.912 | 8.13E-02 |
| nfu00280:Valine, leucine and isoleucine degradation | 5 | 0.760 | 8.19E-02 |
