# Supplementary material for: Life in the fastlane? A comparative analysis of gene expression profiles across annual, semi-annual, and non-annual killifishes (Cyprinodontiformes: Nothobranchiidae)
Source: PLoS One. 2024 Sep 10;19(9):e0308855. doi: 10.1371/journal.pone.0308855 (PMC11386455; doi:10.1371/journal.pone.0308855)
Supplement: S5 Table — Enriched pathways obtained from submitting the DEGs to DAVID webserver. Threshold of minimum gene counts 2 (belonging to an annotation term) and EASE score threshold 0.05 were used to determine significant KEGG pathways. (DOCX) [file pone.0308855.s005.docx]

**S5 Table.** KEGG: non-annuals vs. annuals (liver). Enriched pathways obtained from submitting the DEGs to DAVID webserver. Threshold of minimum gene counts 2 (belonging to an annotation term) and EASE score threshold 0.05 were used to determine significant KEGG pathways.

| **Term** | **Count** | **% from DEGs** | **PValue** |
| --- | --- | --- | --- |
| nfu04310:Wnt signaling pathway | 15 | 2.389 | 0.0055 |
| nfu03015:mRNA surveillance pathway | 9 | 1.433 | 0.0070 |
| nfu04540:Gap junction | 10 | 1.592 | 0.0113 |
| nfu04330:Notch signaling pathway | 8 | 1.274 | 0.0119 |
| nfu00513:Various types of N-glycan biosynthesis | 6 | 0.955 | 0.0191 |
| nfu04114:Oocyte meiosis | 10 | 1.592 | 0.0265 |
| nfu00100:Steroid biosynthesis | 4 | 0.637 | 0.0269 |
| nfu00510:N-Glycan biosynthesis | 6 | 0.955 | 0.0447 |
