# Supplementary material for: Life in the fastlane? A comparative analysis of gene expression profiles across annual, semi-annual, and non-annual killifishes (Cyprinodontiformes: Nothobranchiidae)
Source: PLoS One. 2024 Sep 10;19(9):e0308855. doi: 10.1371/journal.pone.0308855 (PMC11386455; doi:10.1371/journal.pone.0308855)
Supplement: S6 Table — Enriched pathways obtained from submitting the DEGs to DAVID webserver. Threshold of minimum gene counts 2 (belonging to an annotation term) and EASE score threshold 0.05 were used to determine significant KEGG pathways. (DOCX) [file pone.0308855.s006.docx]

**S6 Table.** KEGG: semi-annuals vs. annuals (liver). Enriched pathways obtained from submitting the DEGs to DAVID webserver. Threshold of minimum gene counts 2 (belonging to an annotation term) and EASE score threshold 0.05 were used to determine significant KEGG pathways.

| **Term** | **Count** | **% from DEGs** | **PValue** |
| --- | --- | --- | --- |
| nfu01100:Metabolic pathways | 41 | 11.295 | 1.24E-04 |
| nfu00513:Various types of N-glycan biosynthesis | 5 | 1.377 | 5.10E-03 |
| nfu00630:Glyoxylate and dicarboxylate metabolism | 4 | 1.102 | 1.42E-02 |
| nfu04070:Phosphatidylinositol signaling system | 6 | 1.653 | 1.98E-02 |
| nfu00100:Steroid biosynthesis | 3 | 0.826 | 3.48E-02 |
