# Supplementary material for: Life in the fastlane? A comparative analysis of gene expression profiles across annual, semi-annual, and non-annual killifishes (Cyprinodontiformes: Nothobranchiidae)
Source: PLoS One. 2024 Sep 10;19(9):e0308855. doi: 10.1371/journal.pone.0308855 (PMC11386455; doi:10.1371/journal.pone.0308855)
Supplement: S7 Table — Enriched pathways obtained from submitting the DEGs to DAVID webserver. Threshold of minimum gene counts 2 (belonging to an annotation term) and EASE score threshold 0.05 were used to determine significant KEGG pathways. (DOCX) [file pone.0308855.s007.docx]

**S7 Table.** KEGG: non-annuals vs. semi-annuals (liver). Enriched pathways obtained from submitting the DEGs to DAVID webserver. Threshold of minimum gene counts 2 (belonging to an annotation term) and EASE score threshold 0.05 were used to determine significant KEGG pathways.

| **Term** | **Count** | **% from DEGs** | **PValue** |
| --- | --- | --- | --- |
| nfu04145:Phagosome | 10 | 1.779 | 0.00219 |
| nfu04144:Endocytosis | 12 | 2.135 | 0.02991 |
