# Supplementary material for: Life in the fastlane? A comparative analysis of gene expression profiles across annual, semi-annual, and non-annual killifishes (Cyprinodontiformes: Nothobranchiidae)
Source: PLoS One. 2024 Sep 10;19(9):e0308855. doi: 10.1371/journal.pone.0308855 (PMC11386455; doi:10.1371/journal.pone.0308855)
Supplement: S8 Table — Genes are ranked by the lowest padj value. (DOCX) [file pone.0308855.s008.docx]

**S8 Table.** The top 500 most significant differentially expressed genes in annuals vs. non-annuals. Genes are ranked by the lowest padj value.

| **Gene_ID** | **baseMean** | **log2FC** | **lfcSE** | **stat** | **pvalue** | **padj** |
| --- | --- | --- | --- | --- | --- | --- |
| LOC107387648 | 306.317 | 6.720 | 0.1908 | 29.974 | 2.16E-197 | 3.546E-193 |
| LOC107384484 | 1229.423 | 7.918 | 0.2358 | 29.339 | 3.26E-189 | 2.680E-185 |
| LOC107384930 | 5489.179 | 7.748 | 0.3060 | 22.052 | 9.15E-108 | 5.006E-104 |
| LOC107373195 | 874.595 | 6.153 | 0.2477 | 20.800 | 4.33E-96 | 1.779E-92 |
| LOC107373304 | 46.949 | 6.125 | 0.2607 | 19.661 | 4.63E-86 | 1.521E-82 |
| LOC107382553 | 379.397 | 5.961 | 0.2574 | 19.274 | 8.89E-83 | 2.433E-79 |
| LOC107390481 | 295.039 | 4.986 | 0.2142 | 18.608 | 2.76E-77 | 6.476E-74 |
| farsa | 80.958 | 5.888 | 0.2648 | 18.460 | 4.36E-76 | 8.946E-73 |
| LOC107372906 | 311.561 | 7.995 | 0.3820 | 18.314 | 6.37E-75 | 1.162E-71 |
| LOC107392522 | 122.141 | 5.195 | 0.2319 | 18.094 | 3.58E-73 | 5.873E-70 |
| pecr | 81.336 | 7.333 | 0.3526 | 17.960 | 4.02E-72 | 6.005E-69 |
| LOC107373388 | 862.554 | -9.342 | 0.4738 | -17.606 | 2.22E-69 | 3.043E-66 |
| aldh16a1 | 68.888 | 6.676 | 0.3266 | 17.380 | 1.17E-67 | 1.371E-64 |
| LOC107392516 | 88.903 | 5.751 | 0.2733 | 17.381 | 1.16E-67 | 1.371E-64 |
| eif6 | 53.713 | 6.433 | 0.3179 | 17.092 | 1.69E-65 | 1.854E-62 |
| insig2 | 56.950 | 5.821 | 0.2908 | 16.577 | 1.02E-61 | 1.049E-58 |
| adrm1 | 122.907 | 5.438 | 0.2700 | 16.440 | 9.91E-61 | 9.569E-58 |
| LOC107396651 | 30.223 | 5.033 | 0.2487 | 16.214 | 4.04E-59 | 3.688E-56 |
| chtop | 46.658 | 5.558 | 0.2826 | 16.128 | 1.62E-58 | 1.399E-55 |
| LOC107376444 | 35.625 | 6.061 | 0.3208 | 15.779 | 4.37E-56 | 3.586E-53 |
| LOC107396785 | 148.540 | 3.601 | 0.1656 | 15.706 | 1.38E-55 | 1.075E-52 |
| LOC107372901 | 140.867 | 6.592 | 0.3581 | 15.618 | 5.51E-55 | 4.111E-52 |
| csgr09h1orf50 | 75.728 | 5.520 | 0.2914 | 15.512 | 2.89E-54 | 2.065E-51 |
| LOC107381236 | 226.429 | 6.218 | 0.3399 | 15.352 | 3.45E-53 | 2.361E-50 |
| LOC107384490 | 125.773 | 6.855 | 0.3827 | 15.299 | 7.77E-53 | 5.104E-50 |
| LOC107375463 | 343.474 | 5.897 | 0.3213 | 15.239 | 1.93E-52 | 1.221E-49 |
| LOC107389789 | 23.968 | 5.667 | 0.3090 | 15.104 | 1.52E-51 | 9.223E-49 |
| pck2 | 1092.109 | 5.097 | 0.2730 | 15.007 | 6.65E-51 | 3.900E-48 |
| LOC107379795 | 74.118 | 5.785 | 0.3209 | 14.911 | 2.78E-50 | 1.576E-47 |
| msmb | 767.006 | 7.463 | 0.4378 | 14.763 | 2.53E-49 | 1.387E-46 |
| LOC107382362 | 46.217 | 5.878 | 0.3307 | 14.750 | 3.10E-49 | 1.640E-46 |
| LOC107373109 | 152.721 | 6.685 | 0.3899 | 14.581 | 3.70E-48 | 1.897E-45 |
| mrpl28 | 39.745 | 4.324 | 0.2284 | 14.556 | 5.35E-48 | 2.620E-45 |
| LOC107396079 | 90.472 | 7.834 | 0.4695 | 14.555 | 5.43E-48 | 2.620E-45 |
| LOC107373776 | 160.436 | 6.305 | 0.3695 | 14.356 | 9.82E-47 | 4.607E-44 |
| atox1 | 130.977 | 4.714 | 0.2601 | 14.282 | 2.82E-46 | 1.286E-43 |
| fpgs | 61.961 | 6.568 | 0.3900 | 14.276 | 3.09E-46 | 1.371E-43 |
| mrpl45 | 32.832 | 5.344 | 0.3051 | 14.239 | 5.26E-46 | 2.274E-43 |
| LOC107390461 | 32.976 | 6.441 | 0.3854 | 14.117 | 2.97E-45 | 1.250E-42 |
| nudt5 | 37.871 | 5.339 | 0.3077 | 14.101 | 3.73E-45 | 1.532E-42 |
| ilf2 | 29.778 | 4.782 | 0.2698 | 14.016 | 1.24E-44 | 4.953E-42 |
| LOC107392387 | 32.917 | 5.226 | 0.3034 | 13.928 | 4.29E-44 | 1.678E-41 |
| LOC107377059 | 26.475 | 6.460 | 0.3933 | 13.883 | 8.01E-44 | 3.058E-41 |
| rnasek | 168.734 | 4.757 | 0.2717 | 13.827 | 1.75E-43 | 6.515E-41 |
| agt | 1149.945 | 5.059 | 0.2936 | 13.824 | 1.83E-43 | 6.538E-41 |
| LOC107393561 | 361.188 | 5.325 | 0.3128 | 13.825 | 1.79E-43 | 6.538E-41 |
| mrpl27 | 37.674 | 5.440 | 0.3218 | 13.795 | 2.73E-43 | 9.550E-41 |
| LOC107379601 | 63.325 | 6.041 | 0.3655 | 13.792 | 2.84E-43 | 9.703E-41 |
| LOC107389174 | 736.185 | 6.554 | 0.4033 | 13.769 | 3.92E-43 | 1.314E-40 |
| prdx2 | 1437.588 | 5.334 | 0.3157 | 13.729 | 6.85E-43 | 2.249E-40 |
| LOC107387912 | 27.883 | 5.854 | 0.3542 | 13.704 | 9.61E-43 | 3.094E-40 |
| LOC107386421 | 57.149 | 5.115 | 0.3007 | 13.687 | 1.21E-42 | 3.831E-40 |
| abhd4 | 207.384 | 5.957 | 0.3625 | 13.675 | 1.44E-42 | 4.448E-40 |
| LOC107378962 | 118.939 | 4.746 | 0.2754 | 13.603 | 3.85E-42 | 1.170E-39 |
| slirp | 89.757 | 5.611 | 0.3409 | 13.527 | 1.09E-41 | 3.250E-39 |
| LOC107390430 | 27.431 | 4.434 | 0.2543 | 13.504 | 1.48E-41 | 4.349E-39 |
| phb2 | 90.355 | 4.072 | 0.2283 | 13.455 | 2.89E-41 | 8.312E-39 |
| LOC107381728 | 45.396 | 4.482 | 0.2598 | 13.402 | 5.87E-41 | 1.660E-38 |
| LOC107393345 | 154.735 | 4.705 | 0.2770 | 13.377 | 8.19E-41 | 2.278E-38 |
| gadd45b | 429.753 | 5.954 | 0.3713 | 13.341 | 1.33E-40 | 3.652E-38 |
| psenen | 22.543 | 5.359 | 0.3270 | 13.331 | 1.52E-40 | 4.098E-38 |
| dera | 112.358 | 5.059 | 0.3052 | 13.300 | 2.31E-40 | 6.124E-38 |
| LOC107395994 | 37.033 | 6.767 | 0.4350 | 13.258 | 4.04E-40 | 1.053E-37 |
| afg3l2 | 37.286 | 5.425 | 0.3339 | 13.253 | 4.35E-40 | 1.116E-37 |
| LOC107382558 | 1156.282 | 5.962 | 0.3746 | 13.244 | 4.91E-40 | 1.239E-37 |
| LOC107379855 | 132.396 | 6.148 | 0.3894 | 13.222 | 6.53E-40 | 1.626E-37 |
| LOC107374393 | 20.319 | 6.603 | 0.4254 | 13.171 | 1.28E-39 | 3.144E-37 |
| LOC107386109 | 737.479 | 3.806 | 0.2138 | 13.123 | 2.44E-39 | 5.895E-37 |
| nceh1 | 161.939 | 7.477 | 0.4944 | 13.100 | 3.30E-39 | 7.857E-37 |
| LOC107390794 | 73.435 | 5.579 | 0.3500 | 13.085 | 4.01E-39 | 9.414E-37 |
| LOC107383148 | 505.949 | 6.031 | 0.3846 | 13.083 | 4.14E-39 | 9.446E-37 |
| LOC107383192 | 53.916 | 4.941 | 0.3012 | 13.083 | 4.12E-39 | 9.446E-37 |
| meaf6 | 41.998 | 3.779 | 0.2137 | 13.007 | 1.12E-38 | 2.508E-36 |
| tmbim1 | 25.984 | 6.427 | 0.4178 | 12.989 | 1.41E-38 | 3.130E-36 |
| LOC107389011 | 337.412 | 8.030 | 0.5414 | 12.985 | 1.49E-38 | 3.266E-36 |
| slc38a9 | 34.737 | 5.807 | 0.3706 | 12.971 | 1.80E-38 | 3.884E-36 |
| ghitm | 35.808 | 4.579 | 0.2762 | 12.960 | 2.06E-38 | 4.394E-36 |
| ide | 55.913 | 4.151 | 0.2444 | 12.890 | 5.15E-38 | 1.084E-35 |
| sdccag3 | 23.233 | 5.500 | 0.3500 | 12.859 | 7.66E-38 | 1.592E-35 |
| sdr16c5 | 410.956 | 4.108 | 0.2434 | 12.772 | 2.35E-37 | 4.817E-35 |
| cap1 | 105.671 | 4.338 | 0.2616 | 12.760 | 2.73E-37 | 5.525E-35 |
| LOC107391152 | 71.012 | -6.402 | 0.4238 | -12.748 | 3.21E-37 | 6.423E-35 |
| tgoln2 | 571.926 | 6.281 | 0.4150 | 12.726 | 4.24E-37 | 8.396E-35 |
| LOC107396733 | 80.206 | 7.272 | 0.4930 | 12.722 | 4.44E-37 | 8.674E-35 |
| txn2 | 25.805 | 5.187 | 0.3294 | 12.708 | 5.33E-37 | 1.029E-34 |
| LOC107373864 | 24.662 | 6.167 | 0.4079 | 12.669 | 8.84E-37 | 1.687E-34 |
| LOC107372763 | 29.694 | 6.413 | 0.4288 | 12.625 | 1.54E-36 | 2.905E-34 |
| sptssa | 21.169 | 5.266 | 0.3385 | 12.602 | 2.05E-36 | 3.818E-34 |
| LOC107391206 | 187.335 | 5.834 | 0.3841 | 12.585 | 2.55E-36 | 4.713E-34 |
| cog8 | 50.210 | 5.352 | 0.3466 | 12.556 | 3.67E-36 | 6.692E-34 |
| mrpl34 | 66.720 | 6.927 | 0.4723 | 12.550 | 3.96E-36 | 7.146E-34 |
| psen2 | 29.508 | 5.993 | 0.3981 | 12.542 | 4.38E-36 | 7.814E-34 |
| LOC107374012 | 17.994 | 6.834 | 0.4652 | 12.541 | 4.45E-36 | 7.860E-34 |
| eci2 | 49.075 | 5.645 | 0.3717 | 12.496 | 7.85E-36 | 1.371E-33 |
| csgr06h14orf166 | 17.784 | 5.025 | 0.3234 | 12.446 | 1.48E-35 | 2.557E-33 |
| LOC107380820 | 133.317 | 6.223 | 0.4213 | 12.398 | 2.69E-35 | 4.607E-33 |
| csgr15h1orf174 | 96.294 | 4.349 | 0.2707 | 12.371 | 3.74E-35 | 6.332E-33 |
| LOC107377458 | 653.416 | 3.021 | 0.1634 | 12.365 | 4.03E-35 | 6.752E-33 |
| LOC107384825 | 12.625 | 6.421 | 0.4387 | 12.357 | 4.48E-35 | 7.422E-33 |
| cog4 | 21.929 | 4.970 | 0.3214 | 12.352 | 4.72E-35 | 7.753E-33 |
| LOC107391440 | 80.736 | 6.361 | 0.4350 | 12.324 | 6.75E-35 | 1.097E-32 |
| ncoa4 | 485.227 | 5.128 | 0.3356 | 12.302 | 8.80E-35 | 1.416E-32 |
| slc39a1 | 40.385 | 5.799 | 0.3903 | 12.295 | 9.60E-35 | 1.531E-32 |
| stt3a | 316.420 | 5.278 | 0.3480 | 12.293 | 9.93E-35 | 1.568E-32 |
| slc25a48 | 105.719 | 4.959 | 0.3230 | 12.258 | 1.51E-34 | 2.369E-32 |
| LOC107384147 | 71.207 | 5.455 | 0.3640 | 12.238 | 1.95E-34 | 3.013E-32 |
| LOC107384973 | 43.219 | 3.904 | 0.2377 | 12.213 | 2.65E-34 | 4.074E-32 |
| LOC107383121 | 91.219 | 7.512 | 0.5335 | 12.206 | 2.88E-34 | 4.382E-32 |
| tmem9 | 19.682 | 5.115 | 0.3374 | 12.197 | 3.22E-34 | 4.855E-32 |
| bambi | 30.285 | 7.582 | 0.5412 | 12.163 | 4.91E-34 | 7.328E-32 |
| tmem203 | 13.980 | 4.774 | 0.3111 | 12.130 | 7.29E-34 | 1.079E-31 |
| vma21 | 45.774 | 4.860 | 0.3185 | 12.117 | 8.56E-34 | 1.255E-31 |
| LOC107376526 | 21.401 | 5.333 | 0.3579 | 12.106 | 9.78E-34 | 1.421E-31 |
| LOC107395212 | 15.221 | -7.260 | 0.5173 | -12.102 | 1.04E-33 | 1.492E-31 |
| magt1 | 73.142 | 5.911 | 0.4067 | 12.076 | 1.41E-33 | 2.009E-31 |
| LOC107376634 | 107.787 | 5.257 | 0.3526 | 12.073 | 1.47E-33 | 2.085E-31 |
| LOC107388660 | 51.317 | 4.550 | 0.2949 | 12.038 | 2.25E-33 | 3.150E-31 |
| LOC107373576 | 1645.766 | 8.289 | 0.6068 | 12.011 | 3.12E-33 | 4.329E-31 |
| LOC107374428 | 1945.173 | 5.369 | 0.3637 | 12.010 | 3.14E-33 | 4.329E-31 |
| mgme1 | 45.989 | 4.954 | 0.3294 | 12.006 | 3.30E-33 | 4.515E-31 |
| LOC107389239 | 20.227 | 5.552 | 0.3806 | 11.960 | 5.77E-33 | 7.832E-31 |
| LOC107372902 | 63.910 | 5.078 | 0.3416 | 11.939 | 7.40E-33 | 9.953E-31 |
| LOC107372920 | 19.321 | 4.206 | 0.2686 | 11.938 | 7.50E-33 | 1.001E-30 |
| alg5 | 41.814 | 4.767 | 0.3156 | 11.936 | 7.68E-33 | 1.017E-30 |
| sod1 | 168.593 | 4.243 | 0.2729 | 11.884 | 1.44E-32 | 1.881E-30 |
| wdr45b | 13.371 | 5.940 | 0.4157 | 11.883 | 1.44E-32 | 1.881E-30 |
| LOC107395861 | 9.809 | -7.142 | 0.5181 | -11.855 | 2.02E-32 | 2.616E-30 |
| LOC107374311 | 43.335 | 5.550 | 0.3839 | 11.852 | 2.09E-32 | 2.685E-30 |
| gcsh | 80.298 | -7.531 | 0.5524 | -11.822 | 3.02E-32 | 3.837E-30 |
| LOC107393149 | 45.566 | 6.088 | 0.4310 | 11.806 | 3.63E-32 | 4.587E-30 |
| LOC107381153 | 25.803 | 4.632 | 0.3078 | 11.800 | 3.90E-32 | 4.887E-30 |
| LOC107382359 | 9.715 | 6.105 | 0.4328 | 11.795 | 4.16E-32 | 5.173E-30 |
| comtd1 | 51.681 | 4.746 | 0.3183 | 11.769 | 5.62E-32 | 6.934E-30 |
| galnt11 | 25.524 | 6.230 | 0.4449 | 11.755 | 6.63E-32 | 8.121E-30 |
| txn | 311.224 | 4.144 | 0.2679 | 11.738 | 8.14E-32 | 9.903E-30 |
| LOC107378339 | 35.444 | 5.373 | 0.3730 | 11.725 | 9.50E-32 | 1.139E-29 |
| arl5b | 26.498 | 5.221 | 0.3600 | 11.725 | 9.44E-32 | 1.139E-29 |
| fgfr1op2 | 137.617 | 3.530 | 0.2164 | 11.693 | 1.39E-31 | 1.652E-29 |
| lsm4 | 27.154 | 4.118 | 0.2667 | 11.689 | 1.45E-31 | 1.710E-29 |
| LOC107374421 | 20.684 | 5.417 | 0.3782 | 11.680 | 1.62E-31 | 1.895E-29 |
| clns1a | 16.766 | 5.096 | 0.3510 | 11.671 | 1.78E-31 | 2.078E-29 |
| mrps36 | 47.962 | 4.812 | 0.3267 | 11.666 | 1.89E-31 | 2.187E-29 |
| hsdl2 | 136.196 | 5.031 | 0.3458 | 11.657 | 2.12E-31 | 2.432E-29 |
| vps54 | 41.654 | 5.090 | 0.3527 | 11.597 | 4.25E-31 | 4.850E-29 |
| dpm2 | 22.417 | 6.087 | 0.4390 | 11.587 | 4.77E-31 | 5.404E-29 |
| gldc | 77.110 | 6.131 | 0.4436 | 11.566 | 6.11E-31 | 6.866E-29 |
| LOC107388259 | 11.921 | 5.901 | 0.4239 | 11.563 | 6.34E-31 | 7.080E-29 |
| taz | 16.501 | 5.615 | 0.4002 | 11.533 | 9.04E-31 | 1.003E-28 |
| ppib | 1722.199 | 4.430 | 0.2979 | 11.515 | 1.10E-30 | 1.215E-28 |
| mon1a | 15.053 | 4.516 | 0.3055 | 11.511 | 1.16E-30 | 1.267E-28 |
| LOC107397289 | 18.720 | 5.316 | 0.3752 | 11.503 | 1.28E-30 | 1.390E-28 |
| LOC107384972 | 17.242 | 5.301 | 0.3741 | 11.498 | 1.35E-30 | 1.460E-28 |
| tm4sf4 | 59.664 | 5.765 | 0.4151 | 11.480 | 1.67E-30 | 1.791E-28 |
| slc48a1 | 61.462 | 3.353 | 0.2050 | 11.476 | 1.74E-30 | 1.850E-28 |
| sdhd | 50.197 | 4.702 | 0.3229 | 11.467 | 1.93E-30 | 2.045E-28 |
| cd74 | 90.756 | 4.993 | 0.3487 | 11.453 | 2.28E-30 | 2.397E-28 |
| LOC107386041 | 511.385 | -3.023 | 0.1768 | -11.442 | 2.58E-30 | 2.694E-28 |
| LOC107389947 | 59.031 | 5.408 | 0.3855 | 11.435 | 2.78E-30 | 2.891E-28 |
| ccdc167 | 17.823 | 5.508 | 0.3946 | 11.423 | 3.20E-30 | 3.302E-28 |
| cdc14b | 340.742 | -4.195 | 0.2801 | -11.407 | 3.85E-30 | 3.947E-28 |
| polr1b | 23.450 | 4.520 | 0.3092 | 11.383 | 5.08E-30 | 5.182E-28 |
| churc1 | 12.825 | 4.871 | 0.3408 | 11.358 | 6.75E-30 | 6.841E-28 |
| LOC107373060 | 10.728 | 5.891 | 0.4307 | 11.355 | 7.02E-30 | 7.072E-28 |
| LOC107383530 | 8.548 | 6.003 | 0.4429 | 11.295 | 1.40E-29 | 1.397E-27 |
| LOC107373492 | 27.431 | 6.492 | 0.4876 | 11.265 | 1.96E-29 | 1.948E-27 |
| sf3b5 | 16.042 | 4.833 | 0.3410 | 11.243 | 2.50E-29 | 2.471E-27 |
| pla2g15 | 276.800 | 4.949 | 0.3514 | 11.239 | 2.63E-29 | 2.581E-27 |
| LOC107392521 | 91.068 | 4.879 | 0.3457 | 11.218 | 3.31E-29 | 3.236E-27 |
| kiaa1143 | 32.360 | 3.967 | 0.2649 | 11.199 | 4.14E-29 | 4.020E-27 |
| ap4s1 | 21.128 | 6.436 | 0.4855 | 11.197 | 4.23E-29 | 4.087E-27 |
| LOC107391885 | 50.193 | 6.457 | 0.4880 | 11.184 | 4.91E-29 | 4.711E-27 |
| LOC107385485 | 23.702 | 5.731 | 0.4237 | 11.166 | 6.00E-29 | 5.731E-27 |
| fam96b | 9.879 | 5.324 | 0.3874 | 11.162 | 6.25E-29 | 5.932E-27 |
| angel2 | 27.983 | 3.550 | 0.2285 | 11.159 | 6.48E-29 | 6.111E-27 |
| LOC107382103 | 89.806 | 4.611 | 0.3248 | 11.115 | 1.07E-28 | 9.994E-27 |
| gcat | 37.142 | 4.938 | 0.3547 | 11.101 | 1.24E-28 | 1.154E-26 |
| LOC107396478 | 18.962 | 6.399 | 0.4875 | 11.075 | 1.65E-28 | 1.533E-26 |
| LOC107382594 | 17.138 | 6.392 | 0.4876 | 11.059 | 1.97E-28 | 1.820E-26 |
| LOC107386181 | 50.620 | 5.738 | 0.4285 | 11.058 | 2.00E-28 | 1.820E-26 |
| LOC107386975 | 15.620 | 6.538 | 0.5008 | 11.059 | 1.99E-28 | 1.820E-26 |
| tmem179b | 18.966 | 6.815 | 0.5261 | 11.052 | 2.15E-28 | 1.947E-26 |
| LOC107372608 | 151.010 | 8.667 | 0.6939 | 11.049 | 2.21E-28 | 1.989E-26 |
| sra1 | 18.193 | 4.816 | 0.3455 | 11.045 | 2.31E-28 | 2.069E-26 |
| romo1 | 85.366 | 4.468 | 0.3141 | 11.040 | 2.45E-28 | 2.184E-26 |
| LOC107390851 | 112.125 | 4.575 | 0.3241 | 11.031 | 2.70E-28 | 2.392E-26 |
| lect2 | 5659.316 | 5.173 | 0.3787 | 11.017 | 3.16E-28 | 2.786E-26 |
| LOC107387291 | 35.432 | 4.998 | 0.3630 | 11.012 | 3.35E-28 | 2.941E-26 |
| LOC107390953 | 13.894 | 5.399 | 0.4001 | 10.995 | 4.04E-28 | 3.528E-26 |
| imp4 | 24.995 | 4.235 | 0.2950 | 10.966 | 5.56E-28 | 4.830E-26 |
| rpl7l1 | 37.400 | 6.219 | 0.4766 | 10.949 | 6.70E-28 | 5.792E-26 |
| LOC107380828 | 33.420 | 7.140 | 0.5611 | 10.943 | 7.18E-28 | 6.170E-26 |
| LOC107391502 | 47.290 | 4.035 | 0.2774 | 10.941 | 7.37E-28 | 6.304E-26 |
| trappc1 | 33.685 | 5.318 | 0.3947 | 10.939 | 7.47E-28 | 6.354E-26 |
| parp4 | 39.478 | 5.034 | 0.3688 | 10.937 | 7.68E-28 | 6.495E-26 |
| spcs3 | 349.770 | 4.225 | 0.2950 | 10.933 | 7.99E-28 | 6.727E-26 |
| LOC107374316 | 25.854 | 4.756 | 0.3439 | 10.924 | 8.87E-28 | 7.433E-26 |
| chac1 | 325.556 | 4.983 | 0.3650 | 10.912 | 1.01E-27 | 8.376E-26 |
| pla2g7 | 11.762 | 6.135 | 0.4708 | 10.908 | 1.06E-27 | 8.799E-26 |
| LOC107387931 | 74.427 | 4.865 | 0.3548 | 10.894 | 1.22E-27 | 1.006E-25 |
| LOC107387991 | 12.295 | 6.452 | 0.5004 | 10.894 | 1.22E-27 | 1.006E-25 |
| csgr11h21orf91 | 15.781 | 5.288 | 0.3936 | 10.893 | 1.25E-27 | 1.019E-25 |
| snrpd1 | 29.847 | 4.076 | 0.2824 | 10.890 | 1.28E-27 | 1.041E-25 |
| fuca2 | 11.032 | 5.925 | 0.4525 | 10.885 | 1.36E-27 | 1.099E-25 |
| LOC107392098 | 62.883 | 3.600 | 0.2389 | 10.881 | 1.41E-27 | 1.137E-25 |
| slc30a6 | 55.089 | 5.723 | 0.4341 | 10.880 | 1.43E-27 | 1.148E-25 |
| rgl4 | 191.796 | 3.652 | 0.2441 | 10.865 | 1.70E-27 | 1.352E-25 |
| LOC107384348 | 23.327 | 6.286 | 0.4875 | 10.843 | 2.15E-27 | 1.705E-25 |
| mthfs | 23.125 | 5.813 | 0.4482 | 10.739 | 6.71E-27 | 5.296E-25 |
| ankrd54 | 24.957 | 3.714 | 0.2529 | 10.732 | 7.17E-27 | 5.633E-25 |
| LOC107383203 | 64.485 | 4.356 | 0.3128 | 10.729 | 7.42E-27 | 5.801E-25 |
| npepps | 96.602 | 3.839 | 0.2646 | 10.726 | 7.70E-27 | 5.991E-25 |
| kansl2 | 17.317 | 4.439 | 0.3214 | 10.700 | 1.02E-26 | 7.878E-25 |
| cisd1 | 139.183 | 3.594 | 0.2427 | 10.689 | 1.15E-26 | 8.836E-25 |
| LOC107376754 | 111.323 | 6.231 | 0.4894 | 10.689 | 1.15E-26 | 8.836E-25 |
| ckb | 101.350 | 4.893 | 0.3650 | 10.666 | 1.47E-26 | 1.124E-24 |
| LOC107393048 | 833.139 | 5.383 | 0.4110 | 10.664 | 1.50E-26 | 1.138E-24 |
| rmdn1 | 22.482 | 4.275 | 0.3074 | 10.654 | 1.67E-26 | 1.267E-24 |
| glod5 | 14.252 | 4.864 | 0.3627 | 10.651 | 1.72E-26 | 1.294E-24 |
| LOC107382845 | 83.544 | 4.208 | 0.3019 | 10.627 | 2.23E-26 | 1.673E-24 |
| miox | 101.585 | 5.637 | 0.4374 | 10.603 | 2.89E-26 | 2.160E-24 |
| LOC107382948 | 10.096 | 4.963 | 0.3739 | 10.600 | 2.99E-26 | 2.220E-24 |
| csgr03h7orf55 | 20.398 | 5.401 | 0.4153 | 10.599 | 3.01E-26 | 2.229E-24 |
| ndufaf2 | 24.331 | 3.664 | 0.2513 | 10.598 | 3.04E-26 | 2.237E-24 |
| fcer1g | 26.466 | 5.194 | 0.3959 | 10.596 | 3.12E-26 | 2.288E-24 |
| taf12 | 17.188 | 4.024 | 0.2855 | 10.595 | 3.14E-26 | 2.291E-24 |
| LOC107379074 | 1129.954 | -8.500 | 0.7098 | -10.566 | 4.26E-26 | 3.093E-24 |
| ccndbp1 | 10.117 | 5.539 | 0.4296 | 10.566 | 4.28E-26 | 3.093E-24 |
| ppt1 | 33.647 | 5.434 | 0.4202 | 10.552 | 4.99E-26 | 3.597E-24 |
| LOC107387930 | 86.048 | 6.520 | 0.5232 | 10.550 | 5.08E-26 | 3.641E-24 |
| LOC107394884 | 18.932 | 6.251 | 0.4979 | 10.545 | 5.36E-26 | 3.828E-24 |
| timm21 | 12.469 | 5.117 | 0.3908 | 10.536 | 5.91E-26 | 4.202E-24 |
| mff | 12.686 | 4.185 | 0.3024 | 10.532 | 6.17E-26 | 4.364E-24 |
| fam234a | 52.172 | 3.582 | 0.2455 | 10.516 | 7.32E-26 | 5.155E-24 |
| LOC107388494 | 10.839 | 4.873 | 0.3686 | 10.506 | 8.14E-26 | 5.711E-24 |
| LOC107393356 | 20.719 | 4.888 | 0.3701 | 10.505 | 8.21E-26 | 5.733E-24 |
| triap1 | 10.742 | 5.997 | 0.4761 | 10.495 | 9.09E-26 | 6.326E-24 |
| csgr17h19orf70 | 22.405 | 5.521 | 0.4308 | 10.493 | 9.27E-26 | 6.419E-24 |
| pithd1 | 83.428 | 3.685 | 0.2563 | 10.478 | 1.09E-25 | 7.547E-24 |
| LOC107386128 | 32.278 | 5.768 | 0.4553 | 10.473 | 1.15E-25 | 7.901E-24 |
| srp9 | 15.405 | 5.420 | 0.4222 | 10.468 | 1.21E-25 | 8.283E-24 |
| snapin | 20.488 | 5.517 | 0.4324 | 10.446 | 1.53E-25 | 1.045E-23 |
| lpcat4 | 11.606 | 6.654 | 0.5429 | 10.414 | 2.15E-25 | 1.457E-23 |
| rhbdd1 | 16.448 | 5.959 | 0.4763 | 10.412 | 2.18E-25 | 1.470E-23 |
| mgst3 | 93.798 | 5.566 | 0.4389 | 10.405 | 2.35E-25 | 1.583E-23 |
| LOC107392952 | 1144.025 | 4.202 | 0.3083 | 10.389 | 2.79E-25 | 1.872E-23 |
| slc13a4 | 69.213 | 6.578 | 0.5375 | 10.377 | 3.14E-25 | 2.098E-23 |
| gcdh | 244.531 | 4.809 | 0.3671 | 10.376 | 3.20E-25 | 2.129E-23 |
| slc6a20 | 19.482 | 6.844 | 0.5636 | 10.369 | 3.44E-25 | 2.279E-23 |
| LOC107380824 | 14.007 | 6.447 | 0.5259 | 10.357 | 3.88E-25 | 2.555E-23 |
| taldo1 | 140.303 | 3.638 | 0.2550 | 10.342 | 4.54E-25 | 2.984E-23 |
| LOC107379664 | 93.666 | 5.355 | 0.4218 | 10.326 | 5.35E-25 | 3.500E-23 |
| ndufb4 | 157.045 | 4.012 | 0.2921 | 10.312 | 6.22E-25 | 4.050E-23 |
| dcaf10 | 15.775 | 5.179 | 0.4055 | 10.307 | 6.53E-25 | 4.239E-23 |
| slc43a1 | 315.392 | 4.377 | 0.3279 | 10.300 | 7.04E-25 | 4.554E-23 |
| gng5 | 29.171 | 3.240 | 0.2176 | 10.296 | 7.34E-25 | 4.725E-23 |
| cldn12 | 19.367 | 4.582 | 0.3482 | 10.290 | 7.85E-25 | 5.035E-23 |
| LOC107383147 | 378.283 | 3.941 | 0.2864 | 10.268 | 9.81E-25 | 6.269E-23 |
| bloc1s1 | 15.259 | 4.142 | 0.3063 | 10.257 | 1.11E-24 | 7.039E-23 |
| LOC107373158 | 10.156 | 4.804 | 0.3710 | 10.253 | 1.15E-24 | 7.280E-23 |
| LOC107396771 | 44.717 | -5.360 | 0.4262 | -10.229 | 1.46E-24 | 9.242E-23 |
| csgr12h17orf62 | 18.482 | 6.865 | 0.5739 | 10.220 | 1.61E-24 | 1.014E-22 |
| prdm13 | 6.489 | -6.037 | 0.4929 | -10.218 | 1.65E-24 | 1.037E-22 |
| LOC107391587 | 16.324 | 4.441 | 0.3369 | 10.214 | 1.72E-24 | 1.076E-22 |
| LOC107377840 | 65.836 | 7.045 | 0.5929 | 10.195 | 2.10E-24 | 1.305E-22 |
| LOC107379050 | 13.871 | 4.924 | 0.3850 | 10.192 | 2.14E-24 | 1.321E-22 |
| vamp8 | 33.031 | 3.653 | 0.2603 | 10.192 | 2.15E-24 | 1.321E-22 |
| LOC107395588 | 15.931 | 4.551 | 0.3484 | 10.192 | 2.14E-24 | 1.321E-22 |
| ift88 | 1006.542 | -6.797 | 0.5691 | -10.187 | 2.27E-24 | 1.393E-22 |
| LOC107391533 | 36.296 | 5.312 | 0.4235 | 10.182 | 2.38E-24 | 1.452E-22 |
| xiap | 11.348 | 4.283 | 0.3225 | 10.180 | 2.44E-24 | 1.483E-22 |
| actr10 | 27.399 | 5.015 | 0.3944 | 10.179 | 2.47E-24 | 1.497E-22 |
| psmd10 | 15.719 | 5.130 | 0.4059 | 10.174 | 2.58E-24 | 1.557E-22 |
| LOC107397169 | 124.887 | 4.056 | 0.3010 | 10.154 | 3.18E-24 | 1.914E-22 |
| LOC107391476 | 12.401 | 6.248 | 0.5169 | 10.152 | 3.23E-24 | 1.936E-22 |
| LOC107390373 | 108.485 | 3.415 | 0.2380 | 10.147 | 3.40E-24 | 2.031E-22 |
| ppp2r4 | 5.902 | 5.091 | 0.4033 | 10.145 | 3.48E-24 | 2.069E-22 |
| tomm7 | 63.055 | 4.500 | 0.3450 | 10.145 | 3.49E-24 | 2.069E-22 |
| LOC107392531 | 200.184 | -3.563 | 0.2531 | -10.128 | 4.14E-24 | 2.444E-22 |
| LOC107390654 | 859.721 | -6.774 | 0.5704 | -10.123 | 4.35E-24 | 2.561E-22 |
| cmc1 | 11.281 | 4.008 | 0.2972 | 10.122 | 4.42E-24 | 2.594E-22 |
| gopc | 11.538 | 5.015 | 0.3970 | 10.113 | 4.85E-24 | 2.833E-22 |
| LOC107377748 | 201.159 | 3.198 | 0.2175 | 10.108 | 5.07E-24 | 2.952E-22 |
| ergic1 | 28.672 | 5.062 | 0.4020 | 10.105 | 5.24E-24 | 3.042E-22 |
| gtf2h5 | 18.907 | 5.094 | 0.4052 | 10.105 | 5.27E-24 | 3.045E-22 |
| sri | 12.748 | 5.083 | 0.4042 | 10.102 | 5.41E-24 | 3.119E-22 |
| LOC107389244 | 38.189 | 4.266 | 0.3234 | 10.100 | 5.52E-24 | 3.169E-22 |
| LOC107388308 | 127.876 | 3.487 | 0.2464 | 10.095 | 5.84E-24 | 3.342E-22 |
| LOC107389325 | 124.078 | 4.330 | 0.3299 | 10.094 | 5.87E-24 | 3.346E-22 |
| gpr180 | 10.711 | 5.675 | 0.4634 | 10.088 | 6.24E-24 | 3.543E-22 |
| snrpa1 | 16.067 | 3.613 | 0.2591 | 10.086 | 6.36E-24 | 3.601E-22 |
| cpq | 17.912 | 4.678 | 0.3651 | 10.073 | 7.25E-24 | 4.090E-22 |
| smim20 | 32.483 | 5.324 | 0.4295 | 10.067 | 7.70E-24 | 4.328E-22 |
| LOC107376431 | 9.686 | 5.655 | 0.4625 | 10.065 | 7.88E-24 | 4.411E-22 |
| LOC107394361 | 21.200 | 6.770 | 0.5733 | 10.065 | 7.90E-24 | 4.411E-22 |
| fam63a | 33.710 | 4.038 | 0.3019 | 10.064 | 7.97E-24 | 4.419E-22 |
| LOC107374512 | 18.226 | 3.780 | 0.2762 | 10.064 | 7.97E-24 | 4.419E-22 |
| ankrd9 | 8.437 | 6.186 | 0.5155 | 10.061 | 8.21E-24 | 4.540E-22 |
| LOC107376358 | 217.760 | 3.917 | 0.2903 | 10.050 | 9.15E-24 | 5.039E-22 |
| olah | 10.809 | 6.009 | 0.4985 | 10.047 | 9.47E-24 | 5.199E-22 |
| LOC107380004 | 1170.196 | 4.338 | 0.3324 | 10.041 | 1.01E-23 | 5.509E-22 |
| fbxo34 | 36.927 | 4.656 | 0.3641 | 10.041 | 1.01E-23 | 5.509E-22 |
| tmem19 | 32.439 | 3.977 | 0.2971 | 10.017 | 1.28E-23 | 6.949E-22 |
| LOC107375576 | 2141.904 | 4.450 | 0.3444 | 10.016 | 1.30E-23 | 7.032E-22 |
| psmg2 | 9.312 | 5.103 | 0.4097 | 10.015 | 1.31E-23 | 7.057E-22 |
| LOC107395703 | 70.385 | 6.018 | 0.5012 | 10.012 | 1.35E-23 | 7.245E-22 |
| tkt | 9.010 | -6.227 | 0.5221 | -10.011 | 1.37E-23 | 7.353E-22 |
| znf414 | 12.150 | 4.584 | 0.3582 | 10.006 | 1.44E-23 | 7.689E-22 |
| yars | 35.064 | 5.118 | 0.4117 | 10.000 | 1.52E-23 | 8.084E-22 |
| LOC107386021 | 14.676 | 5.768 | 0.4772 | 9.993 | 1.64E-23 | 8.732E-22 |
| cstf1 | 8.369 | 4.770 | 0.3775 | 9.988 | 1.72E-23 | 9.113E-22 |
| mogat2 | 56.769 | 8.105 | 0.7124 | 9.974 | 1.99E-23 | 1.050E-21 |
| LOC107374810 | 55.453 | 3.836 | 0.2846 | 9.964 | 2.20E-23 | 1.157E-21 |
| LOC107381854 | 44.481 | 5.462 | 0.4480 | 9.960 | 2.27E-23 | 1.191E-21 |
| eya4 | 24.590 | 5.791 | 0.4811 | 9.958 | 2.32E-23 | 1.214E-21 |
| arpc2 | 85.070 | 3.882 | 0.2894 | 9.958 | 2.33E-23 | 1.214E-21 |
| LOC107396835 | 12.490 | 5.106 | 0.4124 | 9.957 | 2.35E-23 | 1.221E-21 |
| banf1 | 38.464 | 3.956 | 0.2969 | 9.956 | 2.38E-23 | 1.231E-21 |
| idi1 | 216.092 | 5.362 | 0.4382 | 9.953 | 2.45E-23 | 1.263E-21 |
| LOC107376617 | 8.957 | 6.287 | 0.5316 | 9.947 | 2.60E-23 | 1.339E-21 |
| tm7sf2 | 331.473 | 4.607 | 0.3629 | 9.940 | 2.79E-23 | 1.430E-21 |
| LOC107379916 | 81.782 | 7.053 | 0.6097 | 9.928 | 3.14E-23 | 1.606E-21 |
| LOC107395905 | 5.134 | 5.544 | 0.4577 | 9.928 | 3.15E-23 | 1.606E-21 |
| slc25a36 | 376.901 | -3.855 | 0.2878 | -9.922 | 3.35E-23 | 1.702E-21 |
| LOC107385565 | 18.850 | 6.093 | 0.5138 | 9.913 | 3.65E-23 | 1.849E-21 |
| f7 | 228.869 | 3.800 | 0.2825 | 9.910 | 3.77E-23 | 1.902E-21 |
| ccs | 21.501 | 4.491 | 0.3525 | 9.905 | 3.95E-23 | 1.987E-21 |
| LOC107376651 | 60.712 | 5.903 | 0.4953 | 9.898 | 4.23E-23 | 2.118E-21 |
| c5 | 2201.160 | 3.957 | 0.2987 | 9.899 | 4.22E-23 | 2.118E-21 |
| aldh2 | 237.255 | 4.039 | 0.3076 | 9.880 | 5.07E-23 | 2.531E-21 |
| LOC107396558 | 16.177 | 4.693 | 0.3739 | 9.877 | 5.24E-23 | 2.609E-21 |
| lnpep | 19.786 | 3.429 | 0.2463 | 9.861 | 6.14E-23 | 3.044E-21 |
| ifrd1 | 223.820 | 3.232 | 0.2270 | 9.832 | 8.21E-23 | 4.058E-21 |
| mrpl9 | 24.069 | 5.104 | 0.4178 | 9.823 | 8.97E-23 | 4.425E-21 |
| LOC107392597 | 208.425 | 4.442 | 0.3507 | 9.814 | 9.78E-23 | 4.795E-21 |
| atpaf1 | 10.474 | 3.916 | 0.2971 | 9.814 | 9.76E-23 | 4.795E-21 |
| ubl7 | 19.867 | 5.100 | 0.4181 | 9.805 | 1.07E-22 | 5.210E-21 |
| LOC107382981 | 3377.590 | 5.512 | 0.4617 | 9.772 | 1.48E-22 | 7.207E-21 |
| LOC107387362 | 460.376 | 4.887 | 0.3980 | 9.766 | 1.58E-22 | 7.657E-21 |
| LOC107373198 | 17.046 | 6.142 | 0.5266 | 9.764 | 1.60E-22 | 7.758E-21 |
| mrpl41 | 15.151 | 4.551 | 0.3637 | 9.762 | 1.65E-22 | 7.944E-21 |
| LOC107393749 | 51.695 | 6.022 | 0.5156 | 9.740 | 2.04E-22 | 9.814E-21 |
| slc38a7 | 13.986 | 5.844 | 0.4983 | 9.722 | 2.43E-22 | 1.167E-20 |
| LOC107389879 | 15.876 | -6.429 | 0.5587 | -9.717 | 2.54E-22 | 1.217E-20 |
| mtx2 | 14.875 | 3.582 | 0.2658 | 9.712 | 2.67E-22 | 1.274E-20 |
| ndufaf1 | 15.605 | 4.413 | 0.3519 | 9.698 | 3.07E-22 | 1.460E-20 |
| LOC107379378 | 49.752 | 6.667 | 0.5847 | 9.692 | 3.25E-22 | 1.542E-20 |
| pdxp | 45.420 | 4.706 | 0.3829 | 9.679 | 3.70E-22 | 1.751E-20 |
| tat | 380.888 | 4.206 | 0.3317 | 9.665 | 4.24E-22 | 2.002E-20 |
| LOC107372523 | 44.737 | 7.206 | 0.6427 | 9.656 | 4.65E-22 | 2.184E-20 |
| rpia | 32.800 | 3.909 | 0.3012 | 9.656 | 4.66E-22 | 2.184E-20 |
| mrps14 | 13.729 | 4.134 | 0.3247 | 9.651 | 4.85E-22 | 2.270E-20 |
| vta1 | 22.613 | 4.198 | 0.3313 | 9.650 | 4.90E-22 | 2.284E-20 |
| LOC107391778 | 7.014 | 5.247 | 0.4405 | 9.640 | 5.43E-22 | 2.527E-20 |
| LOC107386929 | 17.174 | 5.962 | 0.5149 | 9.636 | 5.62E-22 | 2.608E-20 |
| tmem86a | 51.761 | 4.000 | 0.3117 | 9.625 | 6.27E-22 | 2.900E-20 |
| LOC107397332 | 11.240 | 5.983 | 0.5178 | 9.623 | 6.38E-22 | 2.944E-20 |
| LOC107392646 | 55.430 | -2.782 | 0.1852 | -9.619 | 6.62E-22 | 3.045E-20 |
| npm3 | 21.521 | 4.007 | 0.3129 | 9.612 | 7.13E-22 | 3.268E-20 |
| LOC107372319 | 23.253 | 4.431 | 0.3577 | 9.591 | 8.69E-22 | 3.973E-20 |
| afmid | 22.697 | 4.356 | 0.3501 | 9.585 | 9.25E-22 | 4.218E-20 |
| man2b1 | 58.462 | 4.559 | 0.3716 | 9.577 | 1.00E-21 | 4.558E-20 |
| LOC107380201 | 44.458 | 3.686 | 0.2805 | 9.576 | 1.01E-21 | 4.564E-20 |
| gadd45g | 353.149 | 4.269 | 0.3415 | 9.571 | 1.06E-21 | 4.781E-20 |
| LOC107396744 | 8.845 | 6.051 | 0.5281 | 9.564 | 1.13E-21 | 5.105E-20 |
| acbd4 | 14.566 | 6.562 | 0.5825 | 9.549 | 1.31E-21 | 5.897E-20 |
| fam199x | 16.324 | 3.525 | 0.2645 | 9.545 | 1.36E-21 | 6.099E-20 |
| brd9 | 18.062 | 4.205 | 0.3359 | 9.542 | 1.40E-21 | 6.283E-20 |
| LOC107387203 | 22.909 | 4.650 | 0.3829 | 9.533 | 1.53E-21 | 6.827E-20 |
| slc37a4 | 98.685 | 4.118 | 0.3271 | 9.530 | 1.57E-21 | 6.988E-20 |
| naxe | 55.407 | 4.660 | 0.3840 | 9.530 | 1.58E-21 | 6.993E-20 |
| LOC107392596 | 70.250 | 6.805 | 0.6102 | 9.513 | 1.86E-21 | 8.225E-20 |
| LOC107397045 | 109.756 | 4.512 | 0.3693 | 9.509 | 1.93E-21 | 8.524E-20 |
| LOC107378319 | 38.050 | 5.778 | 0.5029 | 9.502 | 2.07E-21 | 9.093E-20 |
| LOC107396370 | 32.373 | 5.027 | 0.4238 | 9.501 | 2.09E-21 | 9.168E-20 |
| paqr3 | 6.871 | 5.370 | 0.4604 | 9.492 | 2.26E-21 | 9.897E-20 |
| LOC107384584 | 7.679 | 5.490 | 0.4731 | 9.490 | 2.30E-21 | 1.006E-19 |
| rab3d | 11.865 | 5.878 | 0.5148 | 9.477 | 2.63E-21 | 1.145E-19 |
| mospd2 | 180.311 | 3.783 | 0.2937 | 9.475 | 2.66E-21 | 1.153E-19 |
| glmn | 21.040 | 5.705 | 0.4965 | 9.475 | 2.67E-21 | 1.156E-19 |
| ngly1 | 7.453 | 4.606 | 0.3806 | 9.473 | 2.71E-21 | 1.173E-19 |
| carhsp1 | 377.313 | 3.470 | 0.2610 | 9.466 | 2.91E-21 | 1.255E-19 |
| fzd6 | 10.955 | 4.572 | 0.3774 | 9.465 | 2.93E-21 | 1.258E-19 |
| LOC107396025 | 11.241 | 6.445 | 0.5753 | 9.464 | 2.96E-21 | 1.268E-19 |
| smim13 | 16.107 | 4.182 | 0.3363 | 9.463 | 3.00E-21 | 1.282E-19 |
| ivd | 55.231 | 3.440 | 0.2582 | 9.450 | 3.40E-21 | 1.449E-19 |
| mmadhc | 6.424 | 5.658 | 0.4932 | 9.444 | 3.59E-21 | 1.529E-19 |
| wfikkn1 | 6.709 | 5.269 | 0.4523 | 9.438 | 3.82E-21 | 1.619E-19 |
| fbxl5 | 10.173 | 3.774 | 0.2941 | 9.433 | 3.99E-21 | 1.686E-19 |
| LOC107383924 | 46.931 | 3.427 | 0.2574 | 9.428 | 4.19E-21 | 1.767E-19 |
| LOC107379519 | 30.948 | 5.022 | 0.4267 | 9.424 | 4.34E-21 | 1.826E-19 |
| LOC107373870 | 1104.759 | 5.164 | 0.4419 | 9.424 | 4.36E-21 | 1.829E-19 |
| shc2 | 29.740 | -5.508 | 0.4787 | -9.419 | 4.55E-21 | 1.906E-19 |
| LOC107390437 | 8.970 | 6.037 | 0.5351 | 9.413 | 4.84E-21 | 2.023E-19 |
| kctd9 | 103.589 | 2.592 | 0.1692 | 9.407 | 5.09E-21 | 2.120E-19 |
| mrps2 | 6.199 | 4.702 | 0.3937 | 9.403 | 5.32E-21 | 2.212E-19 |
| eef1akmt1 | 9.455 | 4.558 | 0.3792 | 9.383 | 6.44E-21 | 2.669E-19 |
| csgr16h14orf1 | 34.593 | 5.975 | 0.5326 | 9.341 | 9.51E-21 | 3.934E-19 |
| LOC107395745 | 19.194 | 5.665 | 0.4996 | 9.339 | 9.75E-21 | 4.024E-19 |
| LOC107374826 | 27.936 | 4.010 | 0.3224 | 9.337 | 9.93E-21 | 4.086E-19 |
| LOC107389546 | 134.976 | 3.963 | 0.3174 | 9.336 | 1.00E-20 | 4.105E-19 |
| bet1l | 11.765 | 5.475 | 0.4793 | 9.335 | 1.01E-20 | 4.118E-19 |
| tspan3 | 46.815 | 3.357 | 0.2529 | 9.319 | 1.18E-20 | 4.800E-19 |
| acat2 | 71.568 | 4.940 | 0.4231 | 9.311 | 1.27E-20 | 5.170E-19 |
| slc25a16 | 7.735 | 4.885 | 0.4173 | 9.308 | 1.30E-20 | 5.284E-19 |
| LOC107384409 | 35.515 | 4.172 | 0.3409 | 9.305 | 1.34E-20 | 5.415E-19 |
| mrps28 | 40.252 | 4.053 | 0.3282 | 9.302 | 1.38E-20 | 5.591E-19 |
| oxld1 | 5.109 | 5.038 | 0.4341 | 9.301 | 1.40E-20 | 5.636E-19 |
| LOC107388361 | 16.466 | 5.472 | 0.4808 | 9.300 | 1.40E-20 | 5.651E-19 |
| LOC107375655 | 11.552 | 5.222 | 0.4541 | 9.297 | 1.44E-20 | 5.796E-19 |
| LOC107393301 | 9.903 | 5.823 | 0.5195 | 9.285 | 1.62E-20 | 6.468E-19 |
| LOC107388220 | 47.732 | 3.001 | 0.2158 | 9.276 | 1.76E-20 | 7.032E-19 |
| aco1 | 81.068 | 3.966 | 0.3200 | 9.269 | 1.88E-20 | 7.506E-19 |
| mrps30 | 17.327 | 4.836 | 0.4140 | 9.265 | 1.95E-20 | 7.748E-19 |
| pin4 | 16.888 | 4.185 | 0.3439 | 9.263 | 1.99E-20 | 7.900E-19 |
| slc43a3 | 44.342 | 5.384 | 0.4735 | 9.258 | 2.08E-20 | 8.210E-19 |
| man2a1 | 32.023 | 4.286 | 0.3552 | 9.252 | 2.19E-20 | 8.655E-19 |
| LOC107391084 | 18.695 | 5.383 | 0.4737 | 9.252 | 2.20E-20 | 8.655E-19 |
| abat | 58.993 | 4.098 | 0.3350 | 9.247 | 2.31E-20 | 9.062E-19 |
| flad1 | 39.019 | 4.671 | 0.3975 | 9.233 | 2.63E-20 | 1.029E-18 |
| hdac6 | 27.330 | 3.554 | 0.2766 | 9.232 | 2.66E-20 | 1.041E-18 |
| tp53inp1 | 109.087 | 5.980 | 0.5399 | 9.224 | 2.87E-20 | 1.118E-18 |
| yipf5 | 17.493 | 4.887 | 0.4215 | 9.222 | 2.92E-20 | 1.135E-18 |
| LOC107382375 | 53.052 | 3.750 | 0.2984 | 9.219 | 3.01E-20 | 1.167E-18 |
| fam210b | 34.193 | 4.022 | 0.3280 | 9.214 | 3.14E-20 | 1.215E-18 |
| herc4 | 32.252 | 3.715 | 0.2947 | 9.211 | 3.25E-20 | 1.254E-18 |
| ndufaf3 | 10.663 | 5.685 | 0.5088 | 9.208 | 3.33E-20 | 1.284E-18 |
| LOC107379423 | 12.001 | 5.618 | 0.5017 | 9.204 | 3.44E-20 | 1.322E-18 |
| LOC107377914 | 29.446 | 6.313 | 0.5777 | 9.197 | 3.68E-20 | 1.413E-18 |
| slc25a34 | 14.856 | 5.459 | 0.4851 | 9.192 | 3.85E-20 | 1.472E-18 |
| slc25a46 | 10.723 | 4.746 | 0.4078 | 9.186 | 4.09E-20 | 1.562E-18 |
| LOC107374340 | 17.214 | -6.335 | 0.5810 | -9.182 | 4.23E-20 | 1.610E-18 |
| marco | 13.810 | 5.426 | 0.4821 | 9.180 | 4.32E-20 | 1.643E-18 |
| tmem205 | 20.065 | 4.929 | 0.4281 | 9.177 | 4.45E-20 | 1.687E-18 |
| use1 | 8.188 | 5.136 | 0.4517 | 9.158 | 5.29E-20 | 2.001E-18 |
| oat | 1481.240 | 4.362 | 0.3673 | 9.154 | 5.47E-20 | 2.066E-18 |
| LOC107396748 | 12.696 | 4.627 | 0.3967 | 9.142 | 6.14E-20 | 2.313E-18 |
| LOC107397381 | 8.978 | 4.987 | 0.4364 | 9.136 | 6.50E-20 | 2.440E-18 |
| LOC107393009 | 25.265 | 5.772 | 0.5225 | 9.134 | 6.59E-20 | 2.472E-18 |
| abhd6 | 22.597 | 6.220 | 0.5715 | 9.133 | 6.63E-20 | 2.481E-18 |
| pycr1 | 69.951 | 4.819 | 0.4184 | 9.128 | 6.98E-20 | 2.597E-18 |
| slc13a3 | 9.051 | 5.936 | 0.5408 | 9.128 | 6.97E-20 | 2.597E-18 |
| ring1 | 12.921 | 3.830 | 0.3101 | 9.126 | 7.12E-20 | 2.643E-18 |
| serhl2 | 9.482 | 4.586 | 0.3935 | 9.112 | 8.05E-20 | 2.978E-18 |
| LOC107393249 | 268.266 | -3.431 | 0.2667 | -9.113 | 8.04E-20 | 2.978E-18 |
| LOC107384413 | 18.819 | 5.484 | 0.4924 | 9.108 | 8.38E-20 | 3.090E-18 |
| LOC107396520 | 5.406 | 4.923 | 0.4310 | 9.103 | 8.77E-20 | 3.228E-18 |
| lsm8 | 30.770 | 4.323 | 0.3651 | 9.101 | 8.92E-20 | 3.277E-18 |
| LOC107387950 | 15.915 | 5.419 | 0.4856 | 9.100 | 9.07E-20 | 3.318E-18 |
| dctn4 | 22.070 | 4.460 | 0.3802 | 9.100 | 9.06E-20 | 3.318E-18 |
| LOC107376753 | 44.311 | -4.093 | 0.3404 | -9.084 | 1.04E-19 | 3.810E-18 |
| shkbp1 | 7.066 | 5.728 | 0.5206 | 9.083 | 1.06E-19 | 3.850E-18 |
| LOC107387371 | 21.972 | 3.736 | 0.3014 | 9.080 | 1.09E-19 | 3.957E-18 |
| LOC107381771 | 8.835 | 5.219 | 0.4657 | 9.061 | 1.29E-19 | 4.689E-18 |
| atp5sl | 9.661 | 3.800 | 0.3090 | 9.060 | 1.30E-19 | 4.697E-18 |
| LOC107374242 | 15.111 | 5.387 | 0.4844 | 9.056 | 1.35E-19 | 4.874E-18 |
| LOC107395531 | 51.031 | 4.649 | 0.4032 | 9.049 | 1.44E-19 | 5.198E-18 |
| jagn1 | 98.946 | 3.063 | 0.2280 | 9.048 | 1.46E-19 | 5.244E-18 |
| tmem160 | 15.806 | 3.651 | 0.2932 | 9.044 | 1.51E-19 | 5.421E-18 |
| LOC107374597 | 9.674 | 5.933 | 0.5457 | 9.038 | 1.59E-19 | 5.684E-18 |
| LOC107396280 | 37.153 | 4.572 | 0.3952 | 9.038 | 1.59E-19 | 5.687E-18 |
| atraid | 23.359 | 5.670 | 0.5169 | 9.034 | 1.65E-19 | 5.877E-18 |
| commd7 | 13.951 | 4.930 | 0.4352 | 9.029 | 1.73E-19 | 6.144E-18 |
| hus1 | 7.368 | 6.092 | 0.5642 | 9.026 | 1.78E-19 | 6.318E-18 |
| tbl1xr1 | 60.930 | 2.557 | 0.1725 | 9.023 | 1.83E-19 | 6.468E-18 |
| nrbf2 | 10.680 | 4.269 | 0.3625 | 9.017 | 1.93E-19 | 6.829E-18 |
| LOC107379890 | 29.026 | 4.134 | 0.3480 | 9.006 | 2.13E-19 | 7.501E-18 |
| LOC107382398 | 21.842 | 5.071 | 0.4525 | 8.996 | 2.34E-19 | 8.216E-18 |
| traf7 | 20.987 | 3.288 | 0.2545 | 8.993 | 2.42E-19 | 8.472E-18 |
| slc7a10 | 190.156 | 5.465 | 0.4966 | 8.991 | 2.44E-19 | 8.552E-18 |
| fastk | 53.579 | 4.655 | 0.4068 | 8.984 | 2.61E-19 | 9.116E-18 |
| zfyve21 | 69.915 | 3.267 | 0.2526 | 8.975 | 2.84E-19 | 9.900E-18 |
| LOC107396670 | 11.595 | 5.229 | 0.4715 | 8.969 | 2.98E-19 | 1.037E-17 |
| LOC107377927 | 18.372 | 4.138 | 0.3506 | 8.953 | 3.47E-19 | 1.206E-17 |
| LOC107380852 | 12.559 | 4.988 | 0.4455 | 8.952 | 3.49E-19 | 1.210E-17 |
| LOC107380883 | 8.299 | 4.653 | 0.4082 | 8.950 | 3.56E-19 | 1.232E-17 |
| stx12 | 16.478 | 3.142 | 0.2401 | 8.923 | 4.55E-19 | 1.570E-17 |
| LOC107377763 | 14.742 | 6.735 | 0.6434 | 8.914 | 4.94E-19 | 1.699E-17 |
| enthd2 | 10.898 | 4.429 | 0.3849 | 8.908 | 5.19E-19 | 1.782E-17 |
| rab21 | 25.045 | 4.141 | 0.3527 | 8.906 | 5.31E-19 | 1.821E-17 |
| LOC107373896 | 56.703 | 5.169 | 0.4684 | 8.901 | 5.55E-19 | 1.899E-17 |
| nosip | 15.724 | 3.369 | 0.2662 | 8.900 | 5.58E-19 | 1.905E-17 |
| LOC107395294 | 7.476 | 4.561 | 0.4004 | 8.893 | 5.94E-19 | 2.022E-17 |
| LOC107389611 | 12.676 | 5.127 | 0.4642 | 8.890 | 6.08E-19 | 2.068E-17 |
| LOC107391150 | 50.809 | -4.987 | 0.4486 | -8.888 | 6.23E-19 | 2.115E-17 |
| setd3 | 14.847 | 4.699 | 0.4164 | 8.883 | 6.51E-19 | 2.205E-17 |
| nup107 | 14.518 | 3.979 | 0.3356 | 8.877 | 6.84E-19 | 2.312E-17 |
| hmgcl | 9.302 | 3.968 | 0.3344 | 8.875 | 6.99E-19 | 2.355E-17 |
| LOC107377157 | 10.744 | 5.802 | 0.5411 | 8.874 | 7.08E-19 | 2.382E-17 |
| st3gal3 | 17.373 | -3.912 | 0.3283 | -8.872 | 7.19E-19 | 2.413E-17 |
| LOC107391030 | 23.499 | 4.132 | 0.3530 | 8.871 | 7.25E-19 | 2.428E-17 |
| nhp2 | 27.954 | 3.597 | 0.2928 | 8.870 | 7.29E-19 | 2.438E-17 |
| phkg2 | 11.967 | 5.066 | 0.4586 | 8.866 | 7.55E-19 | 2.519E-17 |
| LOC107382961 | 103.614 | -4.913 | 0.4419 | -8.855 | 8.36E-19 | 2.784E-17 |
| LOC107388084 | 42.622 | 3.549 | 0.2881 | 8.845 | 9.15E-19 | 3.043E-17 |
| vamp3 | 36.214 | 3.583 | 0.2921 | 8.841 | 9.45E-19 | 3.135E-17 |
| LOC107372392 | 20.876 | 3.108 | 0.2387 | 8.830 | 1.05E-18 | 3.459E-17 |
| piezo1 | 97.179 | 3.170 | 0.2459 | 8.826 | 1.09E-18 | 3.592E-17 |
| aamp | 24.685 | 3.821 | 0.3198 | 8.822 | 1.12E-18 | 3.694E-17 |
| asns | 52.023 | 4.863 | 0.4382 | 8.815 | 1.19E-18 | 3.915E-17 |
