# Supplementary material for: Life in the fastlane? A comparative analysis of gene expression profiles across annual, semi-annual, and non-annual killifishes (Cyprinodontiformes: Nothobranchiidae)
Source: PLoS One. 2024 Sep 10;19(9):e0308855. doi: 10.1371/journal.pone.0308855 (PMC11386455; doi:10.1371/journal.pone.0308855)
Supplement: Attachment — Submitted filename: Response_to_Reviewers.docx [file pone.0308855.s009.docx]

PONE-D-23-41180

Life in the fastlane? A comparative analysis of gene expression profiles across annual, semi-annual, and non-annual killifishes (Cyprinodontiformes:Nothobranchiidae)

PLOS ONE

Journal Requirements:

1. When submitting your revision, we need you to address these additional requirements.

Please ensure that your manuscript meets PLOS ONE's style requirements, including those for file naming. The PLOS ONE style templates can be found at 
<https://journals.plos.org/plosone/s/file?id=wjVg/PLOSOne_formatting_sample_main_body.pdf> and 
<https://journals.plos.org/plosone/s/file?id=ba62/PLOSOne_formatting_sample_title_authors_affiliations.pdf>

2. Thank you for stating in your Funding Statement: 
KRP - Research reported in this publication was supported by an Institutional Development Award (IDeA) from the National Institute of General Medical Sciences of the National Institutes of Health under grant number P2O GM103424-21
The funders did not play any role in the study design, data collection and analysis, decision to publish, or preparation of the manuscript.

Please provide an amended statement that declares all the funding or sources of support (whether external or internal to your organization) received during this study, as detailed online in our guide for authors at <http://journals.plos.org/plosone/s/submit-now>.  Please also include the statement “There was no additional external funding received for this study.” in your updated Funding Statement. 
Please include your amended Funding Statement within your cover letter. We will change the online submission form on your behalf.

3. Thank you for stating the following in the Acknowledgments Section of your manuscript: 
This study was supported by funding to KRP from the Louisiana Biomedical Research Network. Specifically, the research reported in this publication was supported by an Institutional Development Award (IDeA) from the National Institute of General Medical Sciences of the National Institutes of Health under grant number P2O GM103424-21.

We note that you have provided funding information that is not currently declared in your Funding Statement. However, funding information should not appear in the Acknowledgments section or other areas of your manuscript. We will only publish funding information present in the Funding Statement section of the online submission form. 
Please remove any funding-related text from the manuscript and let us know how you would like to update your Funding Statement. Currently, your Funding Statement reads as follows: 
KRP - Research reported in this publication was supported by an Institutional Development Award (IDeA) from the National Institute of General Medical Sciences of the National Institutes of Health under grant number P2O GM103424-21
The funders did not play any role in the study design, data collection and analysis, decision to publish, or preparation of the manuscript.

Please include your amended statements within your cover letter; we will change the online submission form on your behalf.

4. When completing the data availability statement of the submission form, you indicated that you will make your data available on acceptance. We strongly recommend all authors decide on a data sharing plan before acceptance, as the process can be lengthy and hold up publication timelines. Please note that, though access restrictions are acceptable now, your entire data will need to be made freely accessible if your manuscript is accepted for publication. This policy applies to all data except where public deposition would breach compliance with the protocol approved by your research ethics board. If you are unable to adhere to our open data policy, please kindly revise your statement to explain your reasoning and we will seek the editor's input on an exemption. Please be assured that, once you have provided your new statement, the assessment of your exemption will not hold up the peer review process.

5. Please include captions for your Supporting Information files at the end of your manuscript, and update any in-text citations to match accordingly. Please see our Supporting Information guidelines for more information: <http://journals.plos.org/plosone/s/supporting-information>.

**Reviewers' comments:

Reviewer's Responses to Questions**

**Comments to the Author**

1. Is the manuscript technically sound, and do the data support the conclusions?

The manuscript must describe a technically sound piece of scientific research with data that supports the conclusions. Experiments must have been conducted rigorously, with appropriate controls, replication, and sample sizes. The conclusions must be drawn appropriately based on the data presented.

Reviewer #1: Yes

2. Has the statistical analysis been performed appropriately and rigorously?

Reviewer #1: Yes

3. Have the authors made all data underlying the findings in their manuscript fully available?

The [PLOS Data policy](http://www.plosone.org/static/policies.action#sharing) requires authors to make all data underlying the findings described in their manuscript fully available without restriction, with rare exception (please refer to the Data Availability Statement in the manuscript PDF file). The data should be provided as part of the manuscript or its supporting information, or deposited to a public repository. For example, in addition to summary statistics, the data points behind means, medians and variance measures should be available. If there are restrictions on publicly sharing data—e.g. participant privacy or use of data from a third party—those must be specified.

Reviewer #1: Yes

4. Is the manuscript presented in an intelligible fashion and written in standard English?

PLOS ONE does not copyedit accepted manuscripts, so the language in submitted articles must be clear, correct, and unambiguous. Any typographical or grammatical errors should be corrected at revision, so please note any specific errors here.

Reviewer #1: Yes

5. Review Comments to the Author

Please use the space provided to explain your answers to the questions above. You may also include additional comments for the author, including concerns about dual publication, research ethics, or publication ethics. (Please upload your review as an attachment if it exceeds 20,000 characters)

Reviewer #1: The manuscript submitted by Leow and Piller is a descriptive genomic analysis of gene expression profiles from 62 individuals belonging to eleven different Nothobranchiidae species that fall into three different life-histories (annual, semi-annual, and non-annual). The work provides some new information on how gene regulation can change with life-history, with the possibility of studying the genes highlighted in the manuscript in transgenic and gene silencing studies and examining their impact on longevity.

**Reviewer #1: The manuscript submitted by Leow and Piller is a descriptive genomic analysis of gene expression profiles from 62 individuals belonging to eleven different Nothobranchiidae species that fall into three different life-histories (annual, semi-annual, and non-annual). The work provides some new information on how gene regulation can change with life-history, with the possibility of studying the genes highlighted in the manuscript in transgenic and gene silencing studies and examining their impact on longevity. However, it shows also some flaws, the first one being the lack of a deeper discussion on some of the main results, such as the Gene Ontology analysis, as well as the regulation of DNA repair genes. I think that this could be achieved in different ways: creating some heatmaps or pathway visualization to take a further look at the pathways involved in the analysis, as well as boxplots showing the differences in gene expression at the single gene-level.**

We have a DNA repair heatmap (Fig 5) and also have added a subset of single gene level expression boxplots to show the differences in read counts, but the bulk of the DNA repair gene data presented in Tables 2-4.

**Another aspect that I think could be improved is the presentation of the results in the text, especially in the “Results” section: I think that adding a sentence at the end of each paragraph with a summary of the main observation in the biological context that is then explained in the “Discussion” could help in highlighting the importance of each analysis**.

We have added summary sentences to several of the paragraphs in the results section, but have refrained from going into great detail.

**In addition, the selection of DNA repair genes should be deeper explained and introduced, as the related paragraph represents one of the most highlighted results.**

We have added a paragraph in the introduction about DNA repair genes and the McCrae et al paper. Also, we have added a few sentences to the methods section to justify our focus on the DNA repair genes.

**Regarding the “Results” section, the last thing that seemed out of place to me was the logical disposition of the different paragraphs: in particular, I think that the most logical order for the paragraphs would be this one: the PCA analysis, the DEG analysis, the GO and KEGG analyses, and finally the paragraph on DNA repair genes. I think that this type of order would benefit in maintaining the story fluid.**

Done.

**Finally, I have a minor revision, regarding the figures: I would increase their resolution, as it is difficult to read the information contained in them.**

We uploaded the figures into Adobe Photoshop and increased their resolution and re-uploaded them.
